# Troponin Request Indications and Subsequent Cardiac Management in Acute Care: A Retrospective Quality Improvement Project

**DOI:** 10.7759/cureus.119545

**Published:** 2026-11-12

**Authors:** Ashley C Gogna, Harry Harrison, Deanna Yiu

**Affiliations:** 1 Internal Medicine, University Hospitals Coventry and Warwickshire NHS Trust, Coventry, GBR; 2 Intensive Care, University Hospitals Coventry and Warwickshire NHS Trust, Coventry, GBR; 3 Emergency Medicine, Warwick Hospital, Warwick, GBR

**Keywords:** acute care medicine, cardiac troponin i, emergency medical service, high sensitivity cardiac troponin (hs-ctn), quality improvement projects

## Abstract

Introduction

Troponin-I tests are recommended for suspected acute coronary syndrome (ACS), but troponin-I can also be raised in many other conditions. The test is widely available and frequently requested. Results from requests without a clear indication can be difficult to interpret and may lead to further investigations. We looked at the range of indications for troponin-I requests and what happened after an elevated result.

Aim

This quality improvement project (QIP) had two aims: to describe how the documented indication for troponin-I requests related to recorded further investigation or management after a result meeting local rule-in criteria and to assess whether a poster and a teaching session changed the proportion of requests with chest pain or electrocardiogram (ECG) changes (the primary outcome). No numerical target was set in advance.

Methods

This was a retrospective QIP at a single district general hospital using Plan-Do-Study-Act (PDSA) cycles: baseline data collection, a poster (PDSA 1) and a teaching session for emergency department (ED) doctors (PDSA 2). Troponin-I was measured using the Atellica IM High-Sensitivity Troponin-I assay. Patients met local rule-in criteria for a ‘likely myocardial infarction’ if the result was above 120 ng/L, or rose by at least 20 ng/L at two hours. Each request was assigned to one of three symptom-based categories using the documented indication: chest pain or ECG changes, other possible cardiac symptoms, or other presentations. The outcome was recorded as further cardiac investigation or management (from the discharge summary) in those meeting the criteria. Fisher’s exact and chi-square tests were used, and comparisons between cycles were exploratory.

Results

Of 1,048 patients screened, 988 were included (baseline 489, PDSA 1,282, PDSA 2,217). Overall, 674 (68.2%) had chest pain or ECG changes, 167 (16.9%) had other possible cardiac symptoms and 147 (14.9%) had other presentations. In total, 95 patients (9.6%) met local rule-in criteria, and 42 of these (44.2%) had recorded further investigation or management. The proportion of requests with chest pain or ECG changes did not change significantly across the cycles (69.3%, 64.2% and 71.0%; p = 0.21). In exploratory comparisons, the proportion of all patients who met the criteria and had recorded investigation or management rose from 2.7% to 5.7% and 6.0% (p = 0.048 for each versus baseline), although this cannot be attributed to the interventions.

Among those who met the criteria, recorded further investigation or management followed in 38/58 (65.5%) requests with chest pain or ECG changes, 4/22 (18.2%) with other possible cardiac symptoms and 0/15 (0%) with other presentations (chest pain or ECG changes versus the other two groups combined: odds ratio (OR) 15.7, 95% confidence interval (CI) 4.9-50.5).

Conclusions

Among patients meeting local rule-in criteria, recorded further cardiac investigation or management was more frequent in those with chest pain or ECG changes than in those with other presentations. We saw no clear change in requesting after a poster and a single teaching session. These are exploratory findings suggestive of an association only, and future work could evaluate more active approaches, such as a mandatory indication field, alongside balancing measures.

## Introduction

Problem

High-sensitivity cardiac troponin (hs-cTn) is central to the diagnosis, risk stratification and management of patients with suspected acute coronary syndrome (ACS) and is recommended for this purpose by the European Society of Cardiology (ESC) [1]. However, elevated hs-cTn reflects myocardial injury and is not specific to ACS [1,2], and it can be raised by many other conditions [3,4]. Because this test is widely available, it can be requested outside guideline-supported indications [5-7], which may make results harder to interpret and lead to further investigations and use of healthcare resources. Concern about litigation may also contribute to the number of tests requested [8].

Previous work shows that patient selection influences the diagnostic performance and positive predictive value of troponin testing [5]. It is less clear how the indication for testing relates to subsequent cardiac investigation or management after a raised result in routine acute care practice, including in our own hospital. These actions capture only part of the diagnostic value of testing, but they are recorded routinely and give a practical starting point.

In our hospital, we had the impression that the indication for troponin-I requests was not always clear. When this occurred, teams could be uncertain how to interpret the results and therefore refer on to the medical or cardiology teams. The interpretation from these teams was heavily dependent on the clinical context, and results without a clear indication were sometimes disregarded. We therefore reviewed the ESC guidance on the indications for troponin testing and wanted to see how the indications for our requests related to what happened next.

Aim

This project had two aims. The first, observational, was to describe the association between the documented indication for troponin-I requests and subsequent recorded cardiac investigation or management among patients meeting local rule-in criteria.

The second, improvement-focused, was to assess whether a poster and a teaching session changed the proportion of requests with chest pain or electrocardiogram (ECG) changes as the documented indication, a proxy for guideline-supported requesting. This was the primary improvement outcome. No numerical improvement target was set in advance.

## Materials and methods

Setting

This was a retrospective quality improvement project at a single district general hospital. We reviewed patients who had troponin-I testing in acute care settings: the emergency department (ED), ambulatory emergency care (AEC) and the medical assessment unit (MAU). Troponin-I was measured using the Atellica IM High-Sensitivity Troponin-I assay (Siemens Healthineers, Forchheim, Germany), the assay used at our hospital [9]. This report follows the Standards for QUality Improvement Reporting Excellence (SQUIRE) 2.0 guidelines [10].

Design

We used process mapping (Figure 1) to identify the pathways through which troponin-I tests are requested. This showed there were three routes: first, direct assessment in the ED by the ED team; second, patients streamlined to ambulatory care, who were typically deemed lower risk and potentially for discharge; and third, referral to the medical and cardiology teams. We then looked at what happened after these tests, which ranged from direct discharge to further investigations or referral to specialist services. For patients meeting local rule-in criteria, we recorded any further cardiac investigation or management from the discharge summary (see Definitions).

**Figure 1:**
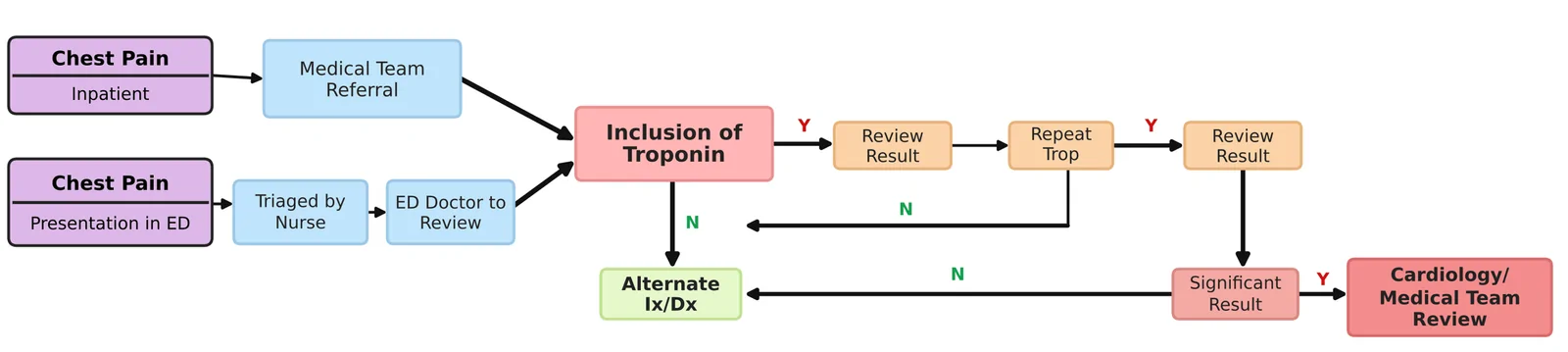
The pathways through which troponin requests were made, directly via the emergency department (ED) team or after referral to the medical team, and the steps that followed.

We also created a driver diagram (Figure 2) to identify factors that influenced whether or not a troponin-I test was requested and used it to help decide which interventions to try.

**Figure 2:**
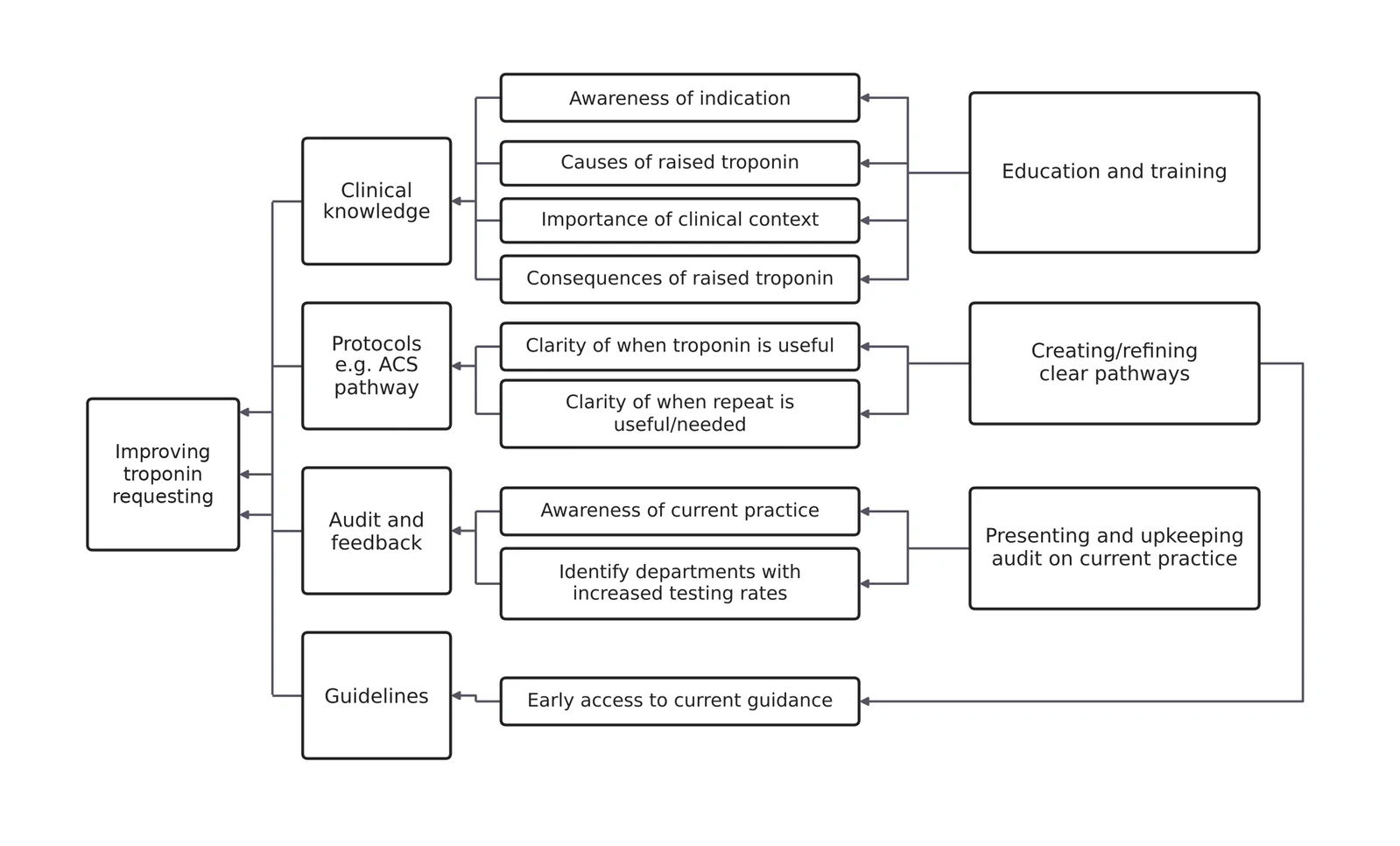
Four drivers of troponin requests and the factors influencing them, including a healthcare practitioner’s clinical knowledge, understanding of trust protocols, use of regular audit and feedback and knowledge of clinical guidelines.

We used the Plan-Do-Study-Act (PDSA) model for quality improvement [11], with four rounds of data collection: two baseline periods before any intervention, then one after the poster (PDSA 1) and one after the teaching session (PDSA 2). The primary improvement outcome was the proportion of requests with chest pain or ECG changes as the documented indication, and no numerical target was set in advance.

Data collection

We asked the biochemistry department to send us the details of all troponin-I tests performed in each collection window, which lasted eight to nine days, so the sample comprised all eligible patients tested in these windows. The timing of the windows was determined by logistical constraints, rather than by a planned schedule. Data were anonymised by assigning a unique identifier to each Medical Record Number (MRN).

Data extraction and unit of analysis

The three authors reviewed the clinical notes and recorded the result values, the presenting symptoms and the indication as documented in the clinical notes. At this first review, we looked only for the indication. Once all four periods had been recorded, the notes of patients meeting local rule-in criteria were reviewed again to find the subsequent management (see Definitions). Indications were therefore recorded for all patients before the outcomes were reviewed. A second independent check or formal disagreement procedure was not used. The unit of analysis was the patient within a collection period. Serial troponin-I tests from the same attendance were combined into one entry per MRN, with all values recorded, and no patient attended more than once within the same collection period. Patients were not matched between periods, so a patient who presented in more than one period was counted separately in each.

Exclusion criteria

We excluded tests performed outside the acute setting (e.g., haematology or oncology outpatient monitoring), requests where no test was performed, tests requested as work-up before a procedure and patients with insufficient documentation. This meant either that no clinical notes had been uploaded to the clinical portal (the most common reason) or that the indication was not recorded or was unclear. These exclusions were made before any outcome information was reviewed, so no patient was excluded because of missing or unclear outcomes. No patients under 16 years old were excluded.

Baseline: data collection 01/10/2024 to 08/10/2024 and 21/04/2025 to 28/04/2025

Baseline data were collected over two eight-day periods, in October and April. We added the April collection to check that requesting had not changed after the resident doctor changeover. The two periods are shown separately in Table 1 and were then pooled for comparison with the intervention cycles.

**Table 1:** Cycle characteristics and outcomes.

Measure	Baseline 1	Baseline 2	Baseline (pooled)	PDSA 1 (poster)	PDSA 2 (teaching)
Patients analysed	229	260	489	282	217
Chest pain or ECG changes, n (%)	171 (74.7)	168 (64.6)	339 (69.3)	181 (64.2)	154 (71.0)
Other possible cardiac symptoms, n (%)	31 (13.5)	50 (19.2)	81 (16.6)	49 (17.4)	37 (17.1)
Other presentations, n (%)	27 (11.8)	42 (16.2)	69 (14.1)	52 (18.4)	26 (12.0)
Meeting local rule-in criteria, n (%)	14 (6.1)	28 (10.8)	42 (8.6)	30 (10.6)	23 (10.6)
Recorded further investigation/management, n (% of all patients)	7 (3.1)	6 (2.3)	13 (2.7)	16 (5.7)	13 (6.0)
Recorded further investigation/management, n (% of those meeting local rule-in criteria)	7/14 (50.0)	6/28 (21.4)	13/42 (31.0)	16/30 (53.3)	13/23 (56.5)

PDSA 1: poster, displayed 05/09/2025; data collection 13/09/2025 to 21/09/2025

The first intervention was an educational poster, ‘Think Before You Trop!’ (see Appendices). Our driver diagram (Figure 2) identified clinical knowledge and awareness of guidelines as a factor in deciding whether to request a troponin-I test. The poster summarised when to request a troponin and when not to, encouraging clinicians to avoid ‘just in case’ requests and to ask whether the result would change management. Posters were placed in the ED, AEC and MAU doctors’ offices and remained on display throughout data collection, which began eight days later using the same methods.

PDSA 2: teaching presentation, delivered 09/12/2025; data collection 12/12/2025 to 19/12/2025

We anticipated that a poster might be ignored, so we planned a more direct intervention for the second cycle: a five-minute presentation to ED doctors at the end of regular departmental teaching, attended by approximately 15 doctors of different grades. It presented the baseline findings, including a breakdown of the requests in the ‘Other presentations’ category (for example, a patient presenting with urinary retention), and discussed barriers to changing practice and ways of improving it. We targeted ED doctors because we believed most requests came through ED rather than AEC or MAU, although the requesting service was not recorded. Data collection began three days later using the same methods. To our knowledge, no local protocols or staffing arrangements changed between the collection periods, apart from the usual four-monthly resident doctor rotations in August, December and April.

Definitions

The 99th percentile upper reference limit for this assay is 45 ng/L overall, with sex-specific limits of 34-39 ng/L for women and 53-54 ng/L for men depending on sample type [9]. Results above this limit indicate myocardial injury, which is not specific to myocardial infarction (MI). We used the local laboratory rule-in criteria, which follow the manufacturer’s 0/2-hour algorithm [9]: a 0-hour result above 120 ng/L, or a rise of at least 20 ng/L at two hours. This algorithm was derived on the ADVIA Centaur System (Siemens Healthineers, Forchheim, Germany) and validated on the Atellica IM Analyzer for suspected non-ST-elevation MI [9,12]. Meeting these criteria identifies patients likely to have an MI, but this is not the same as a confirmed diagnosis, which requires clinical assessment [2]. A result below the threshold is not necessarily normal and does not establish rule-out, so we describe patients as meeting or not meeting local rule-in criteria, and we did not assess rule-out pathways or discharge decisions.

A patient was classed as meeting the criteria if any single result was above 120 ng/L, or if there was a rise of at least 20 ng/L between serial samples. The interval between samples was not recorded, so we could not check that repeat samples were taken at the protocol interval. Patients with a single result were classified on that result alone, and we did not record whether a second sample had been indicated but not taken, so some patients may not have been identified by the rise criterion.

Each request was retrospectively assigned to a symptom-based category according to the indication documented in the clinical notes, using the ESC guideline [1] and clinical context. There is no universally agreed definition of an appropriate troponin request [13], and we did not adjudicate whether each test was clinically justified. The categories therefore describe the documented presenting symptom and should not be read as a judgement of appropriateness. They were defined by the authors, discussed with colleagues and based on the baseline data. They were set after the indications had been extracted but before the requests were counted or analysed, and were applied unchanged to PDSA 1 and PDSA 2: chest pain or ECG changes; other possible cardiac symptoms, including shortness of breath, dizziness, palpitations, arrhythmia or collapse; and other presentations, including sepsis, feeling generally unwell, epigastric pain, reduced exercise tolerance, urinary symptoms, confusion and acute kidney injury.

If a patient had several symptoms, the one most relevant to ACS was used. Chest pain was not further classified by its character. ECG changes (six patients) were a suspected new bundle branch block or T wave inversion, and tachyarrhythmias and bradyarrhythmias were classed as arrhythmias. Indications were taken from the clinical notes for the whole admission, not only those written at the time of the request, so they may not reflect the information available when the test was ordered. Epigastric pain and breathlessness can be presentations of ACS, but it was difficult to establish when this was the clinical suspicion from the documentation, so they were kept separate from the chest pain or ECG changes group.

Further cardiac investigation or management after meeting local rule-in criteria was defined as referral to cardiology, further imaging (echocardiography, CT coronary angiography, cardiac magnetic resonance imaging or invasive angiography), treatment for ACS (including medical management decided by the cardiology team), or revascularisation by percutaneous coronary intervention (PCI) or coronary artery bypass grafting (CABG). We took this from the discharge summary, so the observation period ran from the request until discharge, which ranged from the same day in the ED to an admission of several weeks. An action was counted if it was documented as requested or planned. We did not check that it was completed, review its result, or establish whether it had been planned before the troponin result was available. Cardiac actions in patients not meeting the rule-in criteria and non-cardiac management changes were not collected, and a recorded action does not indicate that it was beneficial.

Statistical analysis

Categorical data are reported as counts and percentages. Two groups were compared using Fisher’s exact test, with the odds ratio (OR) and 95% confidence interval (CI) as the effect size. The three cycles were compared using the chi-square test, reported as χ²(degrees of freedom, N) with Cramér’s V as the effect size. CIs for proportions are exact (Clopper-Pearson). A two-sided p-value below 0.05 was considered significant. No adjustment was made for multiple comparisons, and comparisons between cycles were exploratory.

## Results

Patients and request indications

Data were collected for 1,048 patients. Sixty were excluded (baseline 1: 13, baseline 2: 13, PDSA 1: 14, PDSA 2: 20), leaving 988 patients in the analysis (baseline 1: 229, baseline 2: 260, PDSA 1: 282, PDSA 2: 217). The reasons for exclusion, in the order baseline 1, baseline 2, PDSA 1 and PDSA 2, were haematology or oncology outpatient testing (1, 3, 6 and 11), no clinical notes available (8, 9, 6 and 7), indication not recorded or unclear (4, 1, 1 and 0), work-up for a procedure (0, 0, 1 and 0) and test requested but not performed (0, 0, 0 and 2). Overall, 674 requests (68.2%) had chest pain or ECG changes, 167 (16.9%) other possible cardiac symptoms and 147 (14.9%) other presentations. Table 1 shows these by collection period.

Of the 988 patients, 95 (9.6%) met local rule-in criteria. Of these, 42 (44.2%, 95% CI 34.0-54.8) had recorded further cardiac investigation or management.

Changes across the improvement cycles

The two baseline periods differed. The proportion of requests with chest pain or ECG changes was 74.7% (171/229) in October and 64.6% (168/260) in April (OR 1.61, 95% CI 1.09-2.39, p = 0.018). The proportions meeting local rule-in criteria (6.1% versus 10.8%) and, among those, with recorded action (7/14, 50.0% versus 6/28, 21.4%) also differed, but not significantly (p = 0.08 for each), and numbers were small. Recorded action as a proportion of all patients was similar (3.1% versus 2.3%, p = 0.78).

The proportion of patients meeting local rule-in criteria was similar across the pooled baseline, PDSA 1 and PDSA 2 cycles (8.6%, 10.6% and 10.6%; χ²(2, N = 988) = 1.17, p = 0.56, V = 0.03). Among these patients, recorded further cardiac investigation or management occurred in 31.0%, 53.3% and 56.5% respectively (Table 1). As a proportion of all patients, this rose from 2.7% at pooled baseline to 5.7% after PDSA 1 and 6.0% after PDSA 2 (p = 0.048 for each compared with baseline; p = 1.00 between PDSA 1 and PDSA 2). These comparisons were exploratory, and the borderline p-values do not establish an intervention effect.

The proportion of requests with chest pain or ECG changes did not change significantly across the pooled baseline, PDSA 1 and PDSA 2 cycles (69.3%, 64.2% and 71.0%; χ²(2, N = 988) = 3.15, p = 0.21, V = 0.06), nor did the proportion with other presentations (14.1%, 18.4% and 12.0%; χ²(2, N = 988) = 4.49, p = 0.11, V = 0.07; Figure 3).

**Figure 3:**
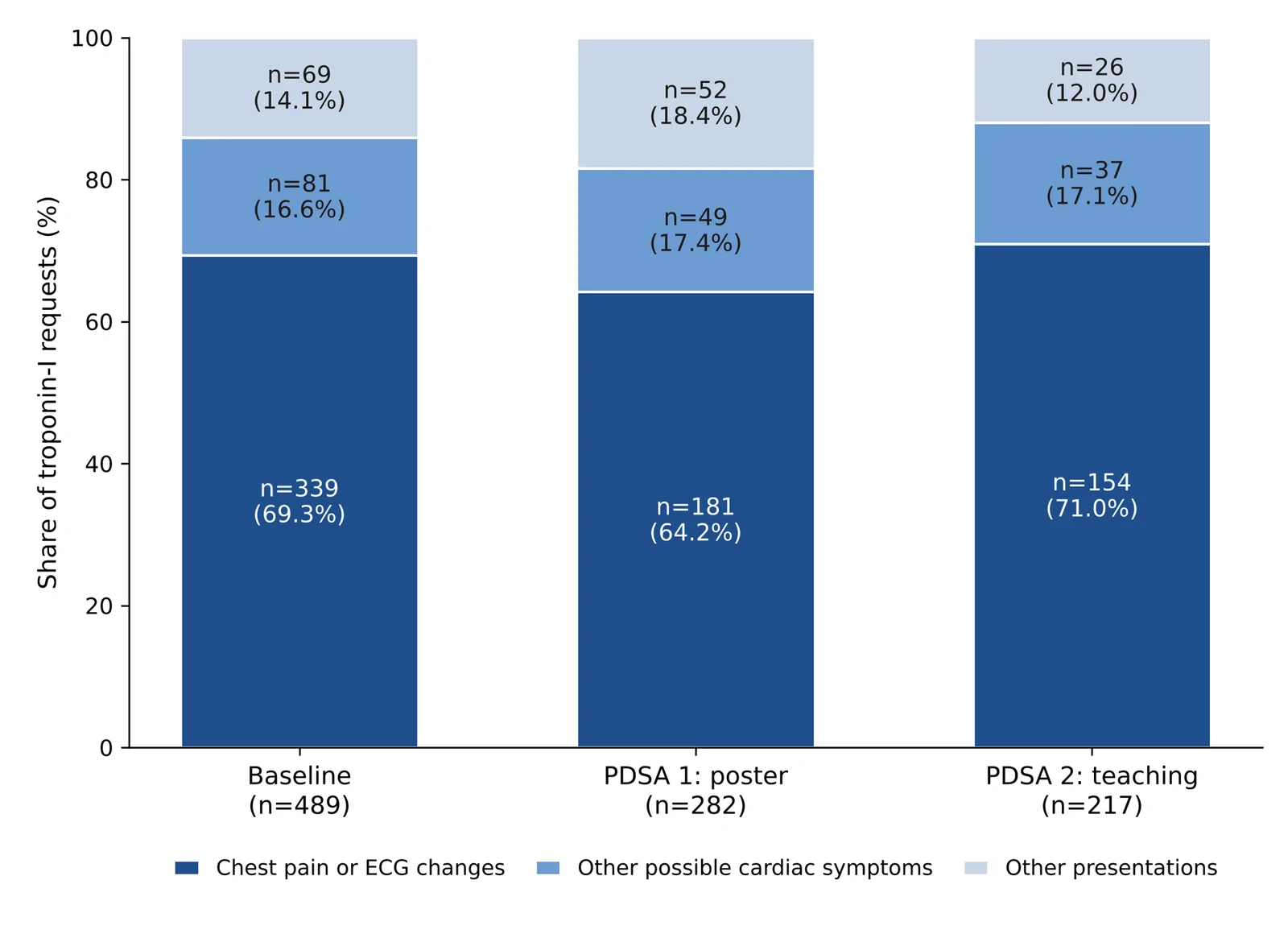
Share of troponin-I request categories by Plan-Do-Study-Act (PDSA) cycle. Bars show the number (n) and percentage of requests in each category; n under each bar is the number of patients analysed in that cycle.

Further investigation or management by indication

Local rule-in criteria were met in 58 of 674 requests with chest pain or ECG changes, 22 of 167 with other possible cardiac symptoms and 15 of 147 with other presentations. Of these, recorded further investigation or management followed in 38 (65.5%, 95% CI 51.9-77.5), four (18.2%, 5.2-40.3) and 0 (0%, 0-21.8), respectively. This was significantly higher for chest pain or ECG changes than for the other two groups combined (38/58 versus 4/37; OR 15.7, 95% CI 4.9-50.5, p < 0.001; Figure 4). The result of 0 of 15 is imprecise, as the true proportion could be as high as 22%. Final diagnoses were not available, so these data should not be interpreted as diagnostic yield.

**Figure 4:**
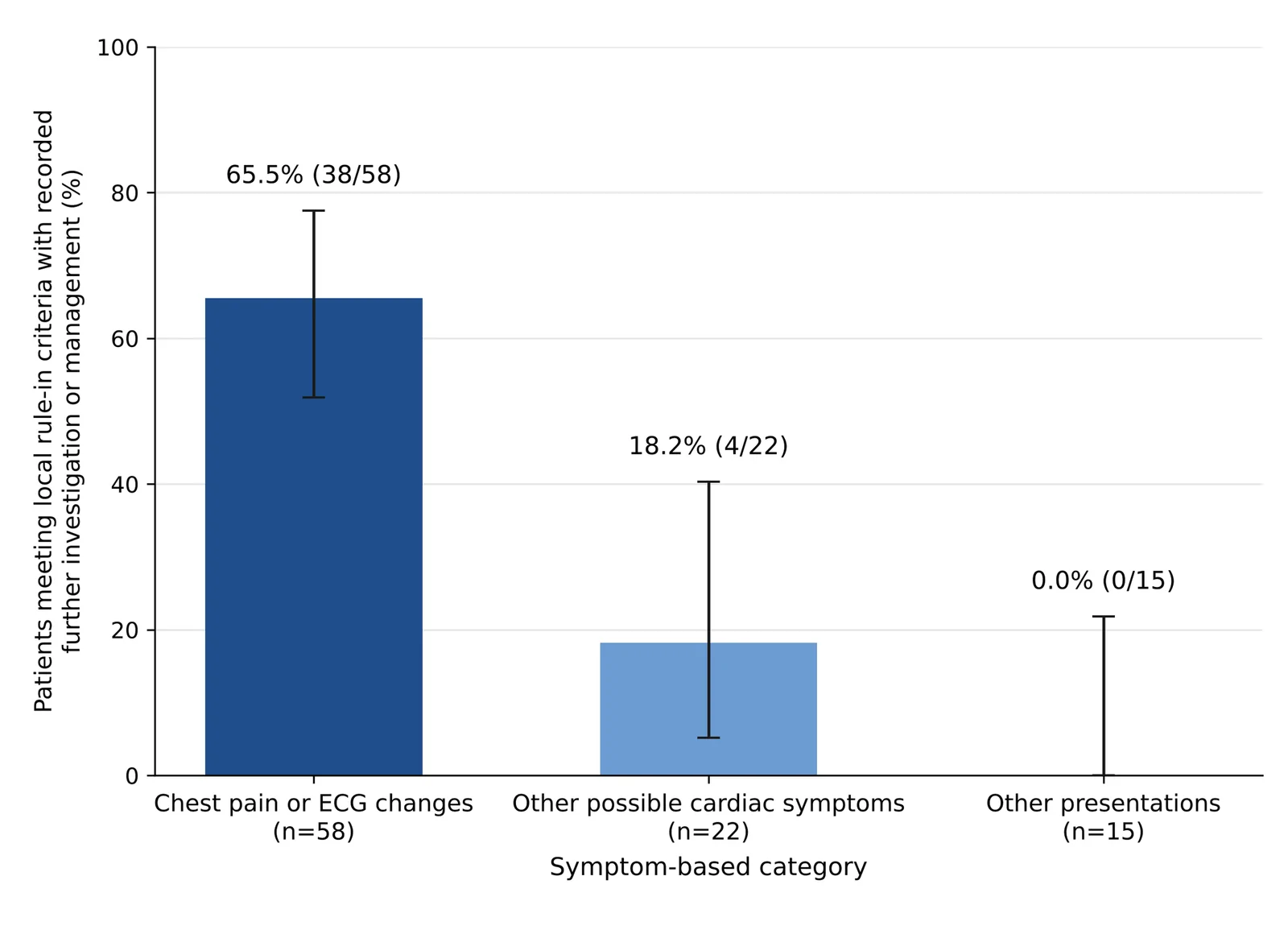
Recorded further cardiac investigation or management by symptom-based category among patients meeting local rule-in criteria (n = 95). Error bars show 95% confidence intervals.

## Discussion

Among patients meeting local rule-in criteria, the symptom-based request category was associated with subsequent cardiac investigation or management. Recorded further investigation or management was most frequent in requests with chest pain or ECG changes (65.5%), and less frequent in those with other possible cardiac symptoms (18.2%) and other presentations (0%) (Figure 4). This is consistent with previous work showing that the interpretation of troponin depends on the clinical context [2,5], and with troponin-I being raised in conditions other than ACS [3,4]. However, this is an association only. The same symptoms used to assign the categories also influence whether clinicians investigate further, so clinical suspicion may explain both the category and the action. Including only patients meeting local rule-in criteria also limits what we can say about all requests, and a lack of recorded action may reflect appropriate conservative care, competing illness, treatment limitations or incomplete documentation rather than an unnecessary test.

We saw no clear change in requesting after the poster and teaching interventions: the proportion of requests with chest pain or ECG changes was 69.3%, 64.2% and 71.0% across the pooled baseline, PDSA 1 and PDSA 2 cycles (Figure 3). There are several possible reasons. A poster is a passive intervention that competes with other posters for attention (see Appendices), and printed educational materials tend to have only small effects on clinical practice [14]. The teaching session was a single session delivered to ED doctors only, so it may not have reached requests made through other teams. Single sessions tend to have limited impact on behaviour [15], particularly where habit, concern about litigation [8] and rotational training also play a part. Data collection began just eight and three days after the interventions, which may have been too soon for any change to show, and we did not collect data afterwards to see whether any change was sustained. The proportion of patients with recorded further investigation or management did rise after the interventions (2.7% at baseline, then 5.7% and 6.0%; Table 1), but as requesting did not clearly change, this may reflect other factors.

We looked only at what happened after a result met local rule-in criteria. Most requests with chest pain or ECG changes did not meet these criteria (616/674, 91%), and the value of testing in these patients was not measured. Results not meeting these criteria cannot be assumed to be normal and do not by themselves exclude MI, which requires appropriate clinical assessment and an assay-specific rule-out pathway [9,12]. Validated rapid rule-out pathways are a diagnostic function of troponin testing that is distinct from prompting action after rule-in, and our outcome does not capture it [1,12]. As ACS was a possible diagnosis in these patients, testing may have been valuable even without further action, although the safety of discharge was not measured. Future work could examine the value of these results in practice.

Our findings do not show which approach would work, but they suggest that more active approaches than a poster or a single teaching session may be worth evaluating. Options include making the indication a required field, repeating teaching at each doctor changeover, and regular audit and feedback, which our driver diagram identified as a potential driver and which has produced small to moderate improvements in practice [16]. A required field would depend less on individual clinicians, but would need to accommodate suspected ACS without chest pain and justified non-ACS testing, for example with a free-text or ‘other’ option. Future cycles could also include balancing measures, such as missed or delayed MI, return visits and time to diagnosis, to help assess the safety of any change in testing.

Troponin testing outside suspected ACS is not necessarily inappropriate. It may also serve a prognostic purpose; for example, in acute pulmonary embolism it is used in risk stratification [17]. A request without a recorded cardiac action may therefore still have been clinically useful, which our outcome, limited to cardiac investigation and ACS treatment, would not capture.

Limitations

Setting and Strengths

This was a single-centre, retrospective project, so the findings may not apply to other settings. It does reflect real-world practice, with 988 patients across two baseline periods and two interventions.

Design and Sampling

Data were collected in short audit periods of eight to nine days, in different months. These cannot establish temporal trends or sustained change, and differences in case mix or staffing between cycles may have affected the results. For logistical reasons, data collection began eight days after the poster was displayed and three days after the teaching session, so the time allowed for each intervention to take effect differed, and longer-term effects were not measured. The two baseline periods, collected six months apart, differed in the proportion of requests with chest pain or ECG changes (74.7% versus 64.6%) without any intervention. This shows that variation of this size can occur between periods, and with no concurrent comparison group, differences between cycles could also have other causes. The project was not designed to detect small differences, so the lack of a significant change does not mean there was none. We recorded only patients who were tested, not the number of acute-care attendances or the requesting service, so the category proportions cannot measure overall test utilisation. Sixty patients were excluded, mostly because notes or an indication were missing, and they may have differed from those included.

Classification and Data Collection

The categories were assigned retrospectively from the documented presenting symptom and do not establish whether a test was clinically justified. A symptom-based approach cannot capture every clinical scenario: for example, chest pain is not invariably an indication for troponin testing; ‘ECG changes’ may include findings unrelated to ischaemia, and epigastric pain and breathlessness can be presentations of ACS, so some requests could reasonably fit more than one category. Indications were taken from the notes for the whole admission, so documentation written after the troponin result may have influenced the classification. We did not reclassify ambiguous cases or repeat the analysis with different definitions, but our main comparison (chest pain or ECG changes versus all other presentations, 38/58 versus 4/37) does not depend on the distinction between the other two groups.

Indications were recorded for all patients before the subsequent management was reviewed, which should limit the influence of outcome knowledge, although the authors were not formally blinded. As in other retrospective chart reviews [18], differential misclassification cannot be excluded, and no independent second check or blinded reclassification of a sample was carried out. A future project could include one.

Outcome and Subgroup

Actions were taken from discharge summaries and counted if documented as requested or planned. We did not confirm that they were completed or review their results, and could not tell whether they had been planned before the troponin result was available. Referral alone does not indicate benefit, and cardiac actions in patients not meeting local rule-in criteria and non-cardiac management changes were not collected, so recorded action is a limited measure of the consequences of testing. Only 95 patients met the rule-in criteria, so subgroup comparisons should be interpreted with caution. The interval between serial samples was not recorded, so some rises may have been missed, and final diagnoses were not available, so the data cannot be interpreted as diagnostic yield.

Future Work

Future work could include balancing measures and examining whether testing less would be safe, whether the recorded actions were beneficial, and what the absence of a recorded action means for the value of a test.

## Conclusions

Among patients meeting local rule-in criteria, recorded cardiac investigation or management was more frequent in those with chest pain or ECG changes than in those with other presentations. We saw no clear change in requesting after the poster and teaching session. These exploratory findings suggest an association only. Future work could examine the value of tests without a recorded action, whether restricting testing would be safe, and whether more active approaches improve requesting.

## Figures and Tables

**Figure 5:**
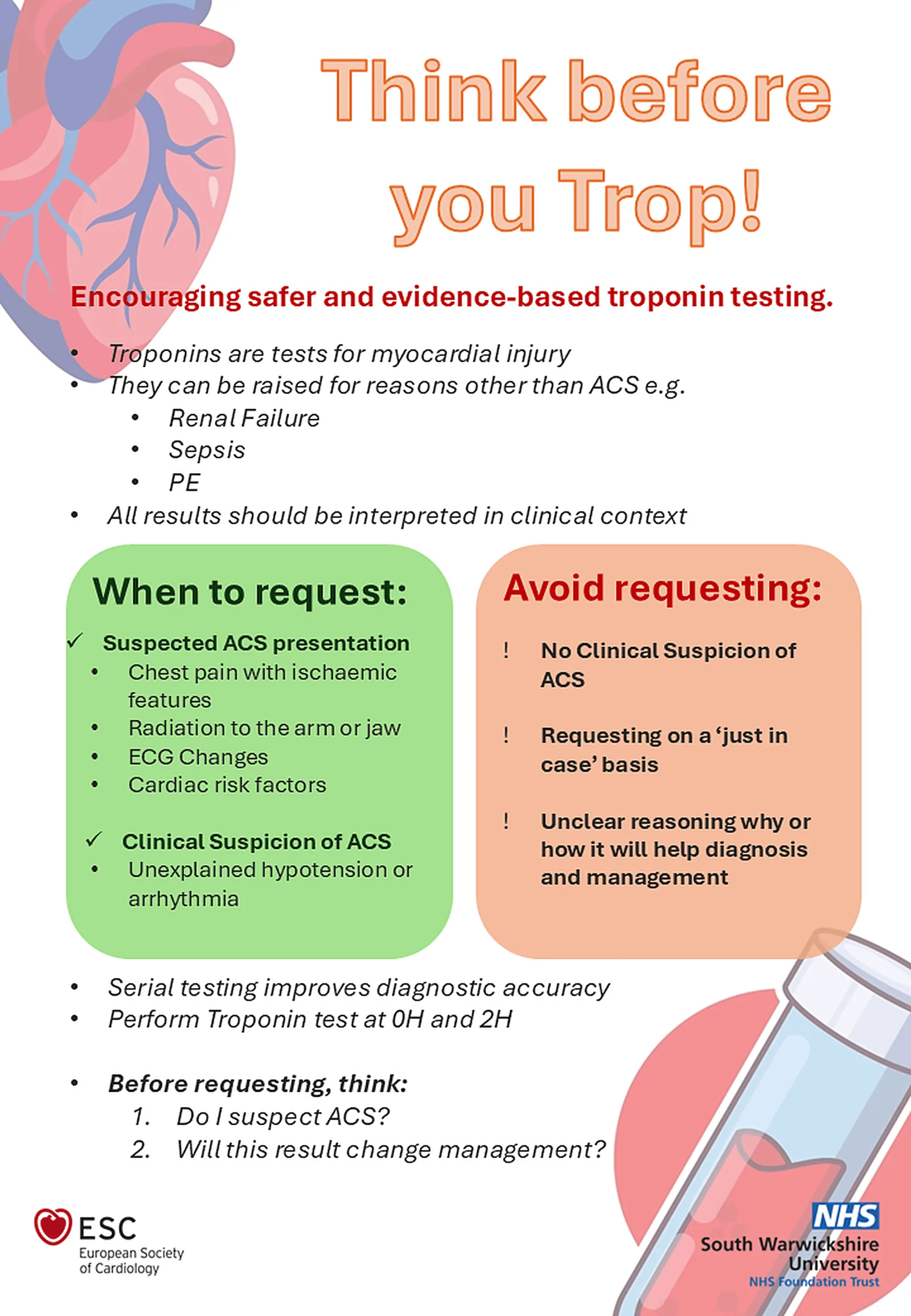
Poster intervention that was placed in the emergency department (ED), medical assessment unit (MAU) and ambulatory emergency care (AEC) for Plan-Do-Study-Act (PDSA) cycle 1. ACS, acute coronary syndrome; PE, pulmonary embolism
